# Data Leakage and Loss in Biodiversity Informatics

**DOI:** 10.3897/BDJ.6.e26826

**Published:** 2018-11-07

**Authors:** A. Townsend Peterson, Alex Asase, Dora Ann Lange Canhos, Sidnei de Souza, John Wieczorek

**Affiliations:** 1 Biodiversity Institute, University of Kansas, Lawrence, United States of America Biodiversity Institute, University of Kansas Lawrence United States of America; 2 University of Ghana, Accra, Ghana University of Ghana Accra Ghana; 3 CRIA, Campinas, Brazil CRIA Campinas Brazil; 4 Museum of Vertebrate Zoology, University of California, Berkeley, United States of America Museum of Vertebrate Zoology, University of California Berkeley United States of America

**Keywords:** biodiversity data, usability, fitness for use, time, place, taxon, informatics, geographic referencing, georeferencing, digitization

## Abstract

The field of biodiversity informatics is in a massive, “grow-out” phase of creating and enabling large-scale biodiversity data resources. Because perhaps 90% of existing biodiversity data nonetheless remains unavailable for science and policy applications, the question arises as to how these existing and available data records can be mobilized most efficiently and effectively. This situation led to our analysis of several large-scale biodiversity datasets regarding birds and plants, detecting information gaps and documenting data “leakage” or attrition, in terms of data on taxon, time, and place, in each data record. We documented significant data leakage in each data dimension in each dataset. That is, significant numbers of data records are lacking crucial information in terms of taxon, time, and/or place; information on place was consistently the least complete, such that geographic referencing presently represents the most significant factor in degradation of usability of information from biodiversity information resources. Although the full process of digital capture, quality control, and enrichment is important to developing a complete digital record of existing biodiversity information, payoffs in terms of immediate data usability will be greatest with attention paid to the georeferencing challenge.

## Introduction

NOTE: responses to longer-form commentaries from reviewers are provided in Suppl. material 1.

Biological diversity is the variety of life on Earth, and provides or sustains, at least in an ultimate sense, all raw materials for human well-being (food, water, shelter). Biodiversity also supports a series of ecosystem services that, although perhaps less tangibly, maintain all natural and human systems (Brauman et al. 2007). Finally, biodiversity constitutes a unique array of lineages reflecting millions of years of evolutionary diversification, such that its preservation is seen as an imperative in and of itself (Wilson 1988), in addition to the intrinsic value of such diversity (Vucetich et al. 2015). However, global biodiversity remains largely undiscovered and undescribed: only 2-20% of species have been described scientifically (Erwin 1991), and knowledge even of the known species remains uneven and irregular, especially across the Tropics.

Primary biodiversity data—i.e., data records that document the occurrence of a particular species at a place at a point in time—represent a central element in the universe of data documenting biodiversity. Primary biodiversity data have many applications, including documenting basic biodiversity patterns (Arita et al. 2008), identifying priority areas for conservation efforts (Loyola et al. 2007), providing baseline information for detection of biotic change (Peterson et al. 2015), and supporting modeling efforts that anticipate biotic responses to local and global change (Kearney et al. 2010). Although the systematics community has long built and maintained information resources on biodiversity, over the past 2-3 decades, availability of and access to such primary biodiversity data records have increased tremendously. Beyond the traditional specimen-based data records, much of this recent growth is thanks to observational data, which includes much-greater numbers of records. Indeed, 1,011,708,045 records are available via the Global Biodiversity Information Facility (GBIF; as of 22 July 2018) alone, thus enabling myriad analyses and summaries to support science and policy (GBIF 2016).

Still, total numbers of primary biodiversity data records that are openly available as digital accessible knowledge (DAK; Sousa-Baena et al. 2013) remain small compared to the universe of biodiversity data that have ever been collected. For instance, via GBIF, in queries as of 22 July 2018, the data portal serves 147,184,231 data records based on specimen documentation; a recent analysis, however, estimated the total number of neontological specimens in existence in world natural history museum and herbarium collections at 1.2-2.1 x 10^9^ specimens (Ariño 2010). Hence, GBIF serves only 6-10% of the specimen-based data held in biological collections, and >90% of specimen-based biodiversity data records remain undigitized or not shared publicly, and not easily available to science and policy applications. Of course, this estimate is based on a single (albeit very large) biodiversity information portal, and other data are not included in this calculation; as such, the actual percentage of specimen data that are digitized and available may be somewhat higher. Estimating the universe of observational (i.e., non-specimen-based) biodiversity data has not been attempted, though clearly far more data exist than are presently available via biodiversity information portals.

**Figure 1.**
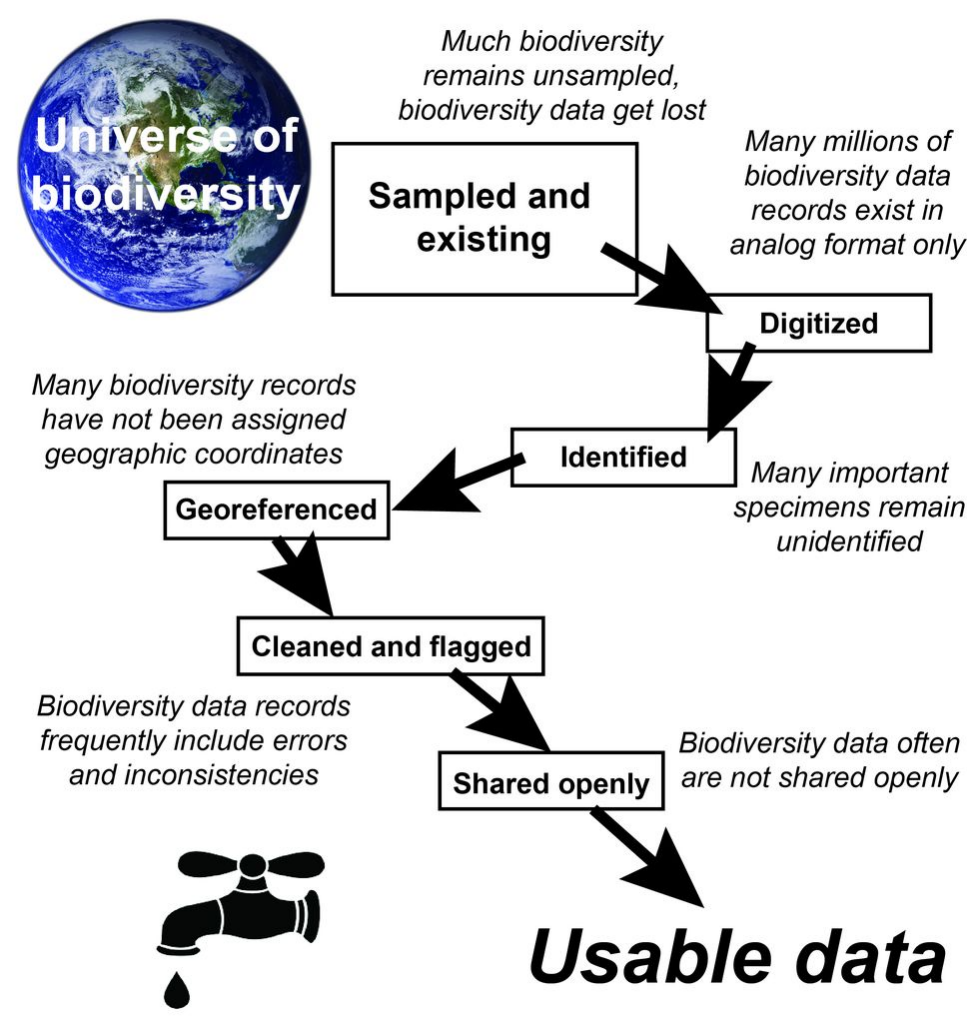
Schematic summarizing the translation between biodiversity and biodiversity data, and how those data “leak,” and get lost and degraded, such that only a small subset is available as usable data for science and policy applications. Note that the particular sequence of steps is not set, and may indeed vary significantly from region to region, taxon to taxon, or source to source.

Even with more than a billion biodiversity specimen and observational data records existing and available in digital format (as of 22 July 2018), many of those records are compromised by missing, partial, or incomplete information, such that they are not usable in many science applications. We term this process as *data leakage*, or data attrition, to emphasize how an initially large data resource is reduced massively via a series of seemingly relatively minor factors (this view of leakage contrasts with a more temporal sequence of degradation or loss; Mesibov 2018). Many important specimens remain with data in analog format only, or are digital, but are unidentified, lack information on date of collection, or lack sufficient information on their geographic provenance. In other cases, digital data lack the key element of geographic coordinates with full documentation of methods and precision of georeferencing. Finally, and perhaps most frustrating, many data records are fully digital and are rich in information, but are not shared. In each case, the effect is the same: data that have been accumulated “leak” out of the main information flow (Fig. 1), and biodiversity information is not in currency for science and policy--this leakage can take the form of data lost owing to failure to capture or preserve infomation at the original moment of specimen collection, error or omission during the data digitization process, or omission because that aspect of the data record has yet to be implemented or prioritized.

In this contribution, we explore the dimensions and magnitude of these data leaks. Using a diverse suite of plant and bird collections as examples, we assess numbers of data records for which information on time, place, and taxon that is missing or incomplete, distinguishing between data that are simply lacking and those that can be added or rescued. We also explore joint effects that relate directly to two typical uses of such data: place x taxon, for ecological niche modelling and species distribution modelling (Peterson et al. 2011), and place x taxon x time, for biodiversity inventory completeness analyses (Asase and Peterson 2016, Ganglo and Kakpo 2016, Wabuyele et al. 2016). Our aim is to reflect on workflows and investment of resources in biodiversity informatics to optimize strategies for building and improving DAK resources. We also see data leakage (attrition) as a phenomenon that exists in any large-scale data infrastructure or analysis, and not only for biodiversity informatics.

## Material and methods

Our analysis sequence is outlined in a protocol file. Briefly, though, we downloaded full institutional datasets for ornithological collections from VertNet (Constable et al. 2010); example datasets were those of the University of Kansas Natural History Museum, Harvard Museum of Comparative Zoology, Slater Museum of Natural History, North Carolina State Museum, Emporia State University, and American Museum of Natural History. Herbarium datasets were downloaded from GBIF (Gaiji et al. 2013); example datasets included Harvard Herbarium, University of Ghana Herbarium, Canadian Museum of Nature, Instituto Nacional de Pesquisas da Amazônia, Museu Goeldi, Michigan State University, University of Arizona, and University of South Florida. Institutional datasets were chosen to span from small to large, representing the diversity of such data, mostly within the United States, but with a few examples from other countries for herbarium data. Our focus in all cases was on species extant or recently extinct, and held in neontological collections of birds and plants, rather than paleontological collections.

Each record from each data set was analyzed with respect to time (i.e., in Darwin Core terms, day, month, year, verbatimEventDate), taxon (genus, subgenus, specificEpithet, infraspecificEpithet, taxonRank), and place (country, stateProvince, county, municipality, locality, verbatimLocality, decimalLatitude, decimalLongitude, coordinateUncertaintyInMeters, coordinatePrecision, verbatimCoordinateSystem, georeferenceProtocol). We evaluated each data record as regards 4 categories of completeness and fitness for use: information missing completely (accorded value 0), information partial (value 1), information incomplete but with sufficient information that it could be “rescued” and brought to completeness (value 2), and information complete and ready for use (value 3). We deemed information as “rescuable” when information can be improved or corrected, such as by georeferencing textual geographic information quantitatively, or by correcting a scientific name that is not a standard name; however, we take a somewhat restrictive view of potential for rescue, in that we do not include as rescuable those specimens that could be reexamined physically to obtain information not in the digital record--rather, we focus on rescue in the sense of the data record per se.

Data on time were considered to be partial when information on day, month, year, or their equivalent in eventDate was missing; time was considered as rescuable when full information appeared to be present in verbatimEventDate, but was not parsed appropriately into day, month, and year, or eventDate. For taxonomic information, names were considered as missing if no genus- or species-level information existed, partial if identified to genus but not to species, and rescuable if not a name listed in at least one taxonomic authority (ornithological authorities checked included Peters 1987, Sibley and Monroe 1990, Clements 2007, and Gill and Donsker 2016). Note that the rescuable/complete distinction was possible only for ornithological data; for plants, no global species names authority lists were available for full digital download (necessary for our assessments), so we considered all full Latin binomials as complete. We note that data from the GBIF data portal are generally expected to be subjected to GBIF taxonomic filtering (Gaiji et al. 2013); however, our experience indicates that the GBIF filters apply to species-based searches, but not to database-level or region-based searches, such that the data analyzed herein have not to our knowledge been subjected to these filters, and indeed included many nonstandard names. For the Brazilian Virtual Herbarium, names were from Brazilian Flora 2020 and Catalogue of Life, in that order. We did not consider the potential for an expert to review and identify the specimen fully as "rescuable," as that step would extend beyond the data to actual handling of the specimen, or at least detailed inspection of images by specialists; although the step of checking the specimen is primordial in the larger picture of biodiversity information management, it is generally very time- and resource-intensive, such that we do not consider it as part of this view of usability and availability of biodiversity information for analyses in short order.

Data on place were considered as missing when geographic coordinates were lacking and textual geographic descriptions lacked information more precise than state. These data were considered as partial when information was available at the level of county/municipality, but not to the level of a specific locality. Data on place were considered as rescuable when the locality was described fully in textual terms, but geographic coordinates missing, or when geographic coordinates were not completely documented with appropriate metadata (Chapman and Wieczorek 2006, Wieczorek et al. 2004). These data were considered as complete only when geographic coordinates were accompanied by full metadata, such that information was present in the fields coordinatePrecision and coordinateUncertaintyInMeters, as this information is crucial to many applications of these data in biodiversity informatics applications, preventing misuse or misinterpretation of coarse-resolution coordinates. We also scored data records as rescuable (not complete) in terms of place when the coordinates were inconsistent—e.g., the coordinate information fell in a country different from that indicated in the data record.

To provide a broader perspective on these data leaks, beyond single datasets, we included overview information parallel to the information for individual datasets for two major, large-scale biodiversity information networks. Specifically, we assessed the Brazilian Virtual Herbarium (5,547,394 records as of 17 February 2017) and VertNet (19,623,087 records as of 17 February 2017). Queries by the information managers of these two networks (authors on this paper) replicated the single-collection analyses described above, to create broad-scale overviews of information completeness across two massive information portals.

For all of the data sets described above, data were summarized in terms of usability for time, taxon, and place separately. We also considered two common applications of primary biodiversity data records. First, for ecological niche modeling and species distribution modeling, a researcher requires information on place and taxon (Peterson et al. 2011), so we inspected joint usability in terms of those two dimensions. For evaluations of inventory completeness, a researcher requires information on time, taxon, and place (Colwell and Coddington 1994), so we assessed usability in those three dimensions jointly. To combine information across multiple dimensions, we took the minimum value of the 4-level categorization given above across the two or three dimensions.

## Data resources

All data analyzed in this study are freely and openly available via online data resources, particularly from VertNet and GBIF. Specific working datasets are available as Suppl. material 3 for birds, and Suppl. material 4 for plants. GBIF downloads correspond to the following digital object identifiers: DOI10.15468/dl.omyjed, DOI10.15468/dl.rii2ou, DOI10.15468/dl.f7nppd, DOI10.15468/dl.gltd7t, DOI10.15468/dl.jreair, DOI10.15468/dl.hwxecn, DOI10.15468/dl.sukiyo, and DOI10.15468/dl.klu2oh

## Results

**Figure 2.**
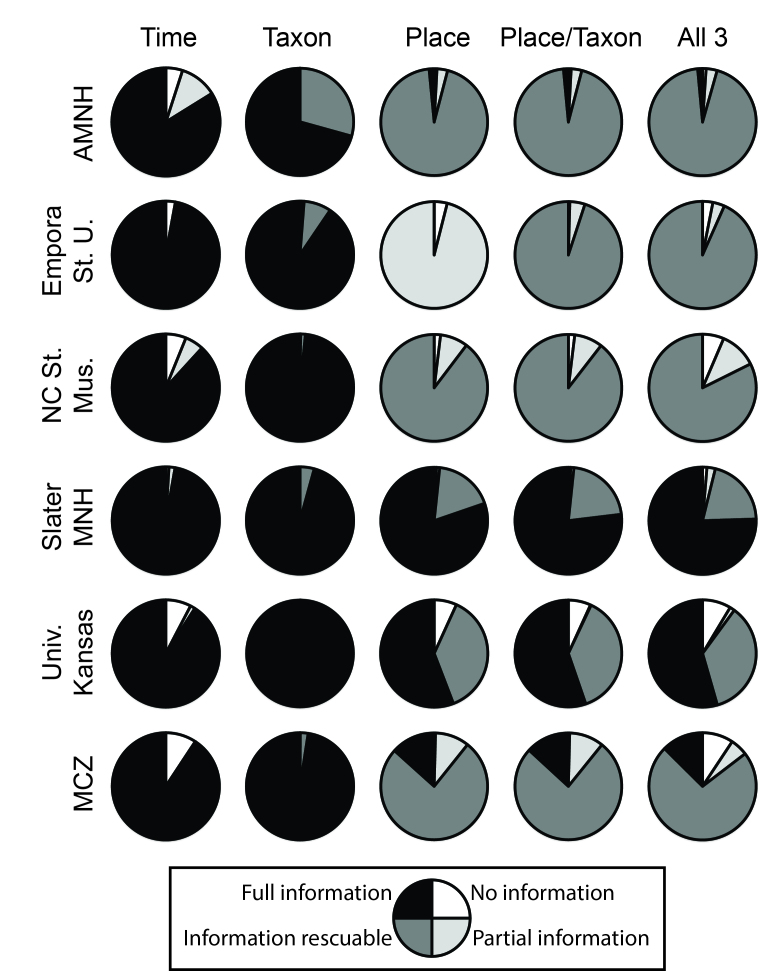
Summary of patterns of completeness and incompleteness of information for 6 ornithological collections, in terms of time, taxon, place, taxon x place, and time x taxon x place.

**Figure 3.**
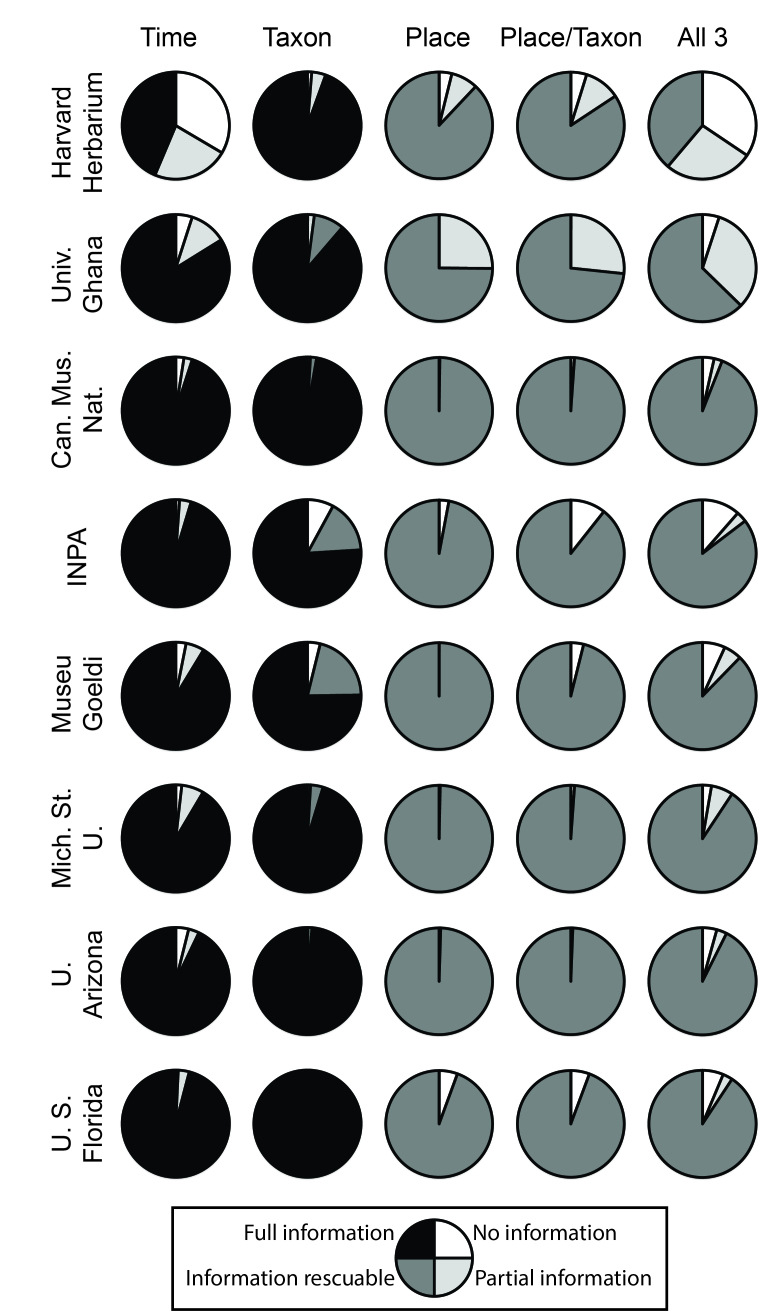
Summary of patterns of completeness and incompleteness of information for 8 herbarium collections, in terms of time, taxon, place, taxon x place, and time x taxon x place. Note that, for lack of a global plant names list that is fully available, we considered rescuable and full taxonomic information together here.

**Figure 4.**
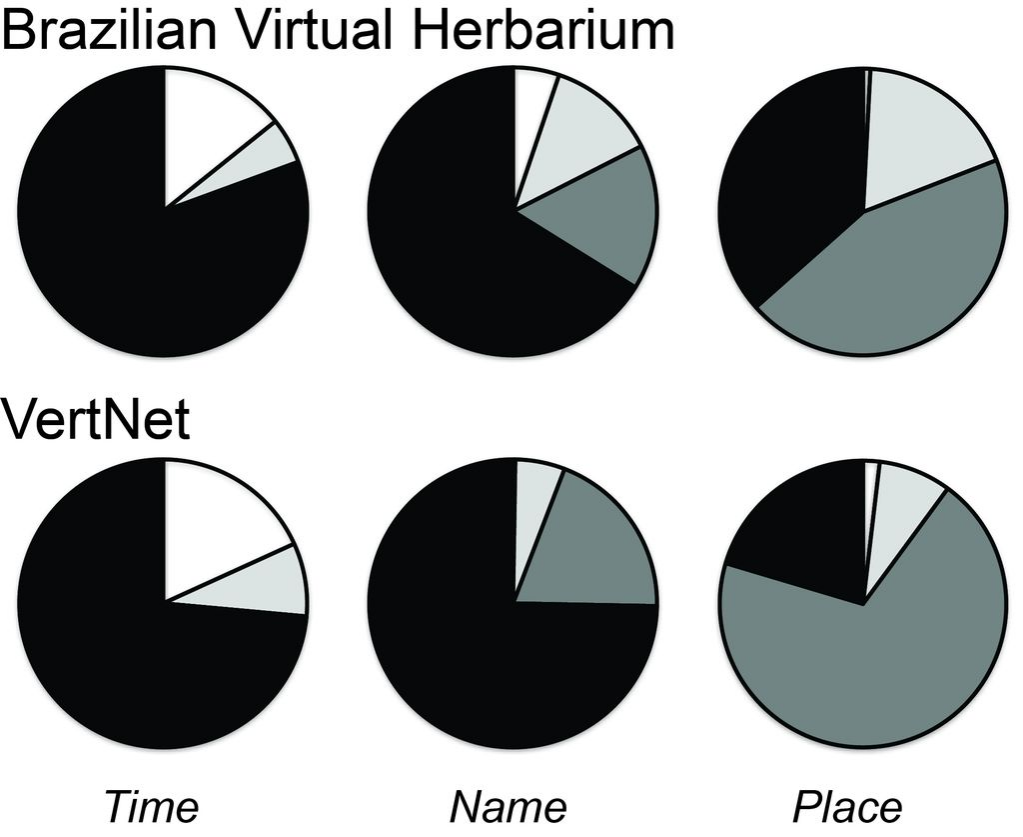
Summary of data leaks in time, place, and taxon information for two major biodiversity informatics initiatives: the Brazilian Virtual Herbarium and VertNet. Note that, for Brazilian Virtual Herbarium, county-level automated georeferencing was included as full georeferencing because it includes information on datum and coordinate uncertainty, although those data records could be georeferenced more finely based on the specific collecting locality. Color scheme follows the key of Figs 2, 3.

Of the three dimensions of the data that we assessed (time, taxon, and place; Figs 2, 3), information regarding time and taxon was most likely to be complete and immediately usable. Taxon was fully usable or rescuable in 98.6% of records for birds, and in 97.3% of records for plants. Time was roughly comparable, being fully usable or rescuable in 94.0% of bird records and 86.2% of plant records (Figs 2, 3). Finally, information on place was least likely to be complete, being fully usable in only 32.4% of bird records and 0% of plant records, and fully usable or rescuable in 78.8% of bird records and 94.2% of plant records. Still, place information was rarely missing entirely (4.5% of records in birds, 1.7% in plants) or incomplete (21.2% in birds, 5.8% in plants), so an important point is that the bulk of records had rescuable information only. These general patterns were similar for the summary information for the Brazilian Virtual Herbarium and VertNet: time and taxon were relatively complete (taxon 74.7% complete for birds, 66.2% complete for plants; time 73.5% complete for birds, 80.6% complete for plants), whereas place was much less well represented by full, analysis-ready information (20.4% complete for birds, 36.6% complete for plants; Fig. 4). A more complete summary of these proportions is provided in Suppl. material 2.

We examined data readiness for use in ecological niche modeling and biodiversity inventory analysis (Figs 2, 3, 4; Table 1). In both cases, place was the most severe constraint on data readiness for use, such that most data were compromised owing to lack of georeferencing of otherwise complete records—these data, however, can be made complete with concerted georeferencing efforts. For inventory analysis, time information completeness reduces data readiness for use still farther, although this constraint was more variable, being major in some cases (e.g., Harvard University Herbarium) and minor in others (e.g., Harvard University Museum of Comparative Zoology).

## Discussion

The analyses presented herein showed that all of the datasets examined suffered some amount of leakage or attrition. That is, for diverse reasons, some information got lost along the way. In some cases, the information loss had occurred at the time of collection of the specimen: i.e., a key data field was not recorded. In such situations, the data record may remain forever without that information. In other cases, however, information loss occurred later, such that some potential exists for rescue and recovery of the information. This potential for rescue with intelligent analysis and hard work is illustrated for the case of date information in a recent analysis (Otegui et al. 2013).

In cases in which the data record may be incomplete, but the data are rescuable, possibilities exist for rapid improvement of DAK resources. For specimen-based biodiversity records, almost always, the specimen can be reexamined and reassessed, perhaps even using new techniques such as DNA barcoding (Pinto et al. 2015); although here we have indicated “rescuable” in a more proximate sense (e.g., correcting a nonstandard Latin binomial), specimen-based records certainly have a greater potential for rescue than observational data, for instance. Although we have focused on specimen-based data in this analysis, the same leakage and loss phenomena affect observational data, albeit not necessarily in the same proportions.

Place information is clearly the dimension in which the greatest need for data rescue exists; that is, biodiversity records almost always hold some spatial information, but the translation of that information into carefully derived and documented geographic coordinates is a complex process (Chapman and Wieczorek 2006, Wieczorek et al. 2004), and often is seen as a step posterior to that of initial data capture (Nelson et al. 2012). The VertNet constellation of projects led this process globally, developing the point-radius method for georeferencing biodiversity data, and implementing large-scale, community-based georeferencing initiatives (Guralnick et al. 2006, Hill et al. 2009, Wieczorek et al. 2004); we note that similar quality standards and flags can and should be implemented for information on time and taxon, to make those data dimensions comparably well documented in comparison with information on place. The VertNet initiative resulted in high-quality, “complete” georeferences for 525,034 distinct locality descriptors and 310,596 unique combinations of longitude and latitude associated with vertebrate specimens, although it is difficult to ascertain to exactly how many specimens these localities correspond.

Indeed, some exploration of place-related data leakage patterns is in order. Of the total of 1,011,708,052 records available via the GBIF data portal as of 22 July 2018, 921,414,317 have geographic coordinates. This total of 91.1% georeferenced is impressive, but is also somewhat deceptive—that is, in the first place, most of those georeferenced records do not include the full metadata to document uncertainty (especially coordinateUncertaintyInMeters), even though this information is crucial to applications such as ecological niche modeling (Anderson et al. 2016). That many niche modelers do not make use of such information does not mean that it is not crucial, but rather that current practice in this field is at times uncareful and incomplete (Peterson 2014). We note that the proportion of records with best-practice georeferencing metadata among specimen-based records was only 52.4% (as of 17 February 2017). These records, nonetheless, represent the crucial historical component of biodiversity information, and thus are indispensable in historical comparisons and detection of change (Peterson et al. 2016).

A further consideration is the interaction between time and data leakage. That is, the specimen record generally is seen as providing the deepest-time view into biodiversity distributions, yet data leakage certainly is more frequent as the age of the specimen increases, as has been documented in previous analyses (Escribano et al. 2016). In many cases, given the greater separation between when data were recorded and the present, these considerations make the data records partial and the leaks irreparable. Changes in technology (e.g., GPS) and data-recording standards can further affect the completeness and utility of older records. Preliminary exploration of the example of the Harvard Herbarium dataset showed greater leakage in older data records in terms of place and time, but less leakage in older data records in terms of taxonomic information; as such, the relationship between time and data leakage appears to be complicated and multidimensional, meriting further research attention. This interaction between age of record and data quality has important implications for the temporal depth of biodiversity information available to the scientific community.

Finally, dimensions of leakage exist that may not be so important for assuring use of the data record. That is, most uses of biodiversity information focus on time, place, and taxon, so other data fields may be less crucial to use of the data in actual analysis; although still important, data sharing and use do not have to await full checking of the full set of fields, as the need for access to such information is immediate and crucial (Pino-Del-Carpio et al. 2014). We make this comment simply to emphasize that dataset perfection is unattainable, and rather that a practical approach should be taken: data records should generally be made available as soon as they are created, just with the assurance that they have sufficient documentation as to not over-represent their precision or importance. That is, for instance, if a temporary georeference is assigned to data records as the centroid of a sizable country, while better and more precise georeferences are developed, that rather imprecise georeference must be accompanied by enough metadata to assure that it is not misinterpreted and misused, or indeed it will be misinterpreted and misused. To repeat, however, perfection will not be attainable in any biodiversity dataset of any size, so we must be practical, and get data online and available globally as soon as is possible.

## Conclusions: the role of e-infrastructures

The explorations presented in this paper lead us to a series of insights into how the field of biodiversity informatics can best move forward towards maximizing its information resources. That is, just investing enormous effort may not be the optimal way forward: rather, “smart” effort may yield much greater pay-offs. Analysis of data leakage, as has been illustrated above, offers ways of thinking about these strategies.

If the goal is to maximize the availability of DAK for analysis and interpretation, one can take into account the sequence of information flow and data leakage (Fig. 1). Fixing leaks late in the sequence will have immediate payoffs in usable information—i.e., if one identifies the final step in the sequence and eliminates that data leak, then all of the data that had not been lost up to that point in the sequence become available for analysis. If, in contrast, one fixes a leak early in the sequence, those data indeed flow farther through the system, but may get lost at some subsequent step before becoming useful to the scientific community. Stated another way, we are in no way downplaying the importance of *de novo *digital capture of biodiversity data, but are only pointing out that the payoff in terms of usable information is greater and more immediate by fixing leaks that occur late in the process, as they flow through immediately to the user in need of the information. Although our emphasis is on a relatively late stage in the digitization workflow, changes to data records must nevertheless be repatriated back to the original data-holder, to avoid creation of conflicts between versions of data records.

This insight can guide time investment in biodiversity informatics initiatives. Analyses such as those we have developed identify immediately the limiting dimensions of DAK usability, thereby focusing immediate investments of time and energy. The clearest signal from our analyses is that detailed and well-documented georeferencing is a crucial aspect of biodiversity informatics, although particular situations can and will differ significantly from this generality. Other insights derive from the data flow and leakage analogy: some biodiversity informatics activities—although important clearly—may not pay off in usable information as immediately. For instance, basic digitization is a major emphasis in the field, and is important for collections management, but digitization in an institutional framework that does not foster data sharing will not improve and increase the availability of information for science and policy. 

In previous analyses and assessments of biodiversity data in biodiversity information portals around the world, the concept of Digital Accessible Knowledge has been proposed and explored (Sousa-Baena et al. 2013). This paper amends and adjusts those ideas—that is, yes, it is crucial that biodiversity data be in digital form, accessible to the broader scientific community, and integrated with other such data as a step towards becoming “knowledge.” However, our analyses in this paper suggest that records being DAK is not sufficient. Rather, here, we illustrate how DAK may nonetheless be compromised by data leakage and loss, to the point that data records are not used in analyses. *Usable* DAK (“UDAK”?) records will be digital, accessible, and integrated, but also will be sufficiently checked, documented, and enriched, so that they are immediately usable in diverse biodiversity informatics analyses. UDAK is conceptually close to the idea of "fitness for use" that has seen considerable discussion recently for biodiversity data (Veiga et al. 2017); both UDAK and fitness for use can best be conceived as contingent on the use to which the data will be put, rather than a single, static quality of the data record. 

Finally, these data leakage phenomena are not in any way unique to specimen-based biodiversity data. Observation-based biodiversity data, which are becoming massively numerous, have their own leaks, such as misidentifications, which create irreparable problems in records; observational data, nonetheless, may not suffer from some of the major leaks that affect specimen data, such as inconsistent taxonomies, given controlled vocabularies in data entry portals. Recent years have seen the assembly of large-scale data resources from heterogeneous sources: e.g., GenBank, and GLOBIS-B. These data infrastructures must reconcile different formats and norms, which at times results in some data records being unusable or less useful in particular analyses. As such, data leakage is not unique to biodiversity data, but rather a general consequence of data sets becoming large.

## Supplementary Material

Supplementary material 1 Response to longer-form reviews Data type: text
Brief description: This file offers detailed responses to two reviewers' comments, which were presented as very long, multipoint comments on the manuscript.
File: oo_222832.pdf ATP

Supplementary material 2 Appendix: Summary Tables Data type: Text and tables
Brief description: These data are the summaries of data leaks in each of three dimensions for each of the bird and herbarium datasets that are depicted in Figures 2 and 3.
File: oo_224908.pdf A. Townsend Peterson

Supplementary material 3 Data for bird collections from VertNet Data type: Primary biodiversity data
Brief description: Comma-delimited ASCII data corresponding to specimens held in a series of museum collections
File: oo_224919.zip A. Townsend Peterson

Supplementary material 4 Herbarium specimen data derived from GBIF Data type: Primary biodiversity data
Brief description: Comma-delimited ASCII data corresponding to herbarium specimens in several collections
File: oo_224921.zip A. Townsend Peterson
