# Supplementary material for: Data Leakage and Loss in Biodiversity Informatics
Source: Biodivers Data J. 2018 Nov 7;(6):e26826. doi: 10.3897/BDJ.6.e26826 (PMC6235996; doi:10.3897/BDJ.6.e26826)
Supplement: Supplementary material 1 — Response to longer-form reviews [file bdj-06-e26826-s001.pdf]

To the Editor: The bulk of the comments offered by the reviewers were as single-point comments in the text, and we have responded to those comments as "responses" on their comments, and then the comment resolved. However, two of the reviewers made extensive comments in single comments… we felt that our responses would be clearest if we treated those longer summaries of many comments in a separate document. We have removed neutral text, or summaries of what the paper does, and distill the reviewers' comments down to single points to which we can respond more clearly.

Vincent Smith

This is an interesting and thought-provoking manuscript covering some important issue. It is also especially timely given the volume of data now available through GBIF and related portals. But I concur with the balance of reviewer opinion that it requires major revision. There are a number of inconsistencies in the arguments made which make it unclear precisely what is being argued for, conflation on some key issues (e.g. data quality and openness associated with digitisation)

> *We have amended and revised the paper extensively to respond to the reviewers' comments and suggestions, such that we hope that the Editor now sees it as ready to proceed to publication.*

… and as one reviewer notes, it is not clear from the text whether we should be most concerned with people being too liberal in their outputs or too conservative in how we process data inputs.

> *This point is a bit nuanced. We are arguing that we can identify key aspects of the biodiversity informatics data flow, and that fixing those key aspects will yield a maximum of available and usable data in the shortest term. We have now adjusted the rhetoric, and we hope that the point will now be clearer. Postel's Law is interesting here. It would seem to be wise … if you are going to publish biodiversity data, you do your best to assure quality. But if you are going to use biodiversity data from diverse sources, you need to take all of the data that you can get, so long as you are confident in its correct interpretation. So yes, Postel's Law applies here, but I am not certain that it helps to develop better data parsers… it simply emphasizes the quandary.*

Overall, this makes for a frustrating read which highlights some important points makes little advance in how to address them. Given these inconsistencies, I fear that aspects of the text could be selectively quoted that might appear to argue for quite different positions. The reviewers make a number of very constructive remarks to improve the manuscript and I would ask they you play close attention to these in the revision. In particular, if you can address the inconsistencies and perhaps highlight some key conclusions (maybe repeated in the abstract) to minimise any ambiguity, I think this would much improve the text and make for a timely publication on an important topic.

> *Again, we hope that our revisions will prove satisfactory re these concerns.*

Rod Page

It has an inconsistent notion of quality, requiring confidence estimates for georeferencing, but not for temporal or taxonomic data.

> *Quite the contrary, we are creating qualitative confidence estimates for temporal and taxonomic data; the MaNIS protocol people already created a metadata schema for georeferencing, and we have used that as a basis for our confidence estimates. The georeferencing metadata are highly developed, but we translate them into our simple, four-level classification. For temporal and taxonomic information, although no detailed metadata schema is available, we explore them for consistency (e.g., April 31st, missing day or month information, nonstandard taxon name, missing specific epithet), and apply our four-level classification as well. So, to be honest, we are treating all three data dimensions the same, but we just have a better-developed data infrastructure for geospatial information. We have included some verbiage to this point in the manuscript, for clarity.*

A number of the conclusions are not fully developed, and it makes statements that deserve more elaboration (e.g., where data gets fixed). In summary, I do not think this manuscript merits publication in its present form.

> *We have taken advantage of three solid reviews to this manuscript, and we have made extensive revisions. We hope that the manuscript is now more meritorious of publication.*

I think it could be improved if it provided more detail on the properties of data in the "rescuable" category, and more specifically how we can reduce the amount of data in this category. For example, does the issue lie with GBIF being unable to parse information provided to it, or are the data providers sending poorly structured data to GBIF? Should we be concerned with people being too liberal in their outputs or too conservative in how they process inputs (cf. Postel's law https://en.wikipedia.org/wiki/Robustness_principle). Can we use the rescuable data to help develop better data parsers?

> *In the first place, we do not see GBIF as the only actor here, either in the sense of being the only data provider, or in the sense of being the only possible actor in the solution. Rather, this is a broad universe of diverse players … scientists and academics, data managers, taxon-based communities, GBIF, etc. … and any one or several of them can and may take on these challenges.*
>
> *Postel's Law is interesting here. It would seem to be wise … if you are going to publish biodiversity data, you do your best to assure quality. But if you are going to use biodiversity data from diverse sources, you need to take all of the data that you can get, so long as you are confident in its correct interpretation. So yes, Postel's Law applies here, but I am not certain that it helps to* develop *better data parsers… it simply emphasizes the quandary, and particularly as it applies to two different actors (data providers* versus *data users).*
>
> *So the question is how to take on the "rescuable" category strategically. We have argued that the best approach is to invest resources immediately in fixing the most distal rescuable data… that is, in the flow of information from original specimen collection through all of the steps of the biodiversity informatics flow-through, to the actual data user, fixing the final stages of that flow will result in more data becoming immediately usable. Of course, that does not mean that one does not collect more specimens, or that*

one does not digitize existing specimens, but only that the step that will pay off most concretely immediately is that of georeferencing. We have revisited the Discussion to make these ideas and concepts more clear.

The notion of "leak" doesn't seem to apply, to me it conjures up information being present and then lost along the way, whereas what seems to be happening is either (a) there is no information present, or (b) it's in a format that makes it difficult to process. It would be different, say, if the providers had supplied georeferenced data, precise dates, or taxonomic names that were lost during the aggregation process (see Bob Mesibov's recent paper https://doi.org/10.3897/zookeys.751.24791 for examples of this).

We use the term leakage to refer to a flow of data from the moment in which the specimen was collected to the user's computer when the data will be put to use. We have found this analogy to plumbing and the flow of water to be quite useful. Indeed, the information did *exist, if nothing else at the moment at which the specimen was collected… the place was obvious, the day was obvious, and the taxon could be ascertained unambiguously. In some sense, however, those data elements "leaked" at some point subsequently, and those bits of information were lost from the overall information flow. That is the sense in which we like the term flow. Regardless, we have cited the Mesibov reference in the introduction, and have avoided now the use of the term "loss," and we have added the term "attrition," in an effort to make the name more palatable.*

The authors are coy about the implications of fixing data closer to its final use. At face value this means that intermediate portals like VertNet and Brazilian Virtual Herbarium are potentially obstacles to access to the best data, and we should eschew such regional or taxonomic portals in favour of sending all data straight to GBIF and fixing the data there. Is this what the authors are arguing?

This comment goes much deeper than we are willing to go. We see potential for data improvement to occur at any of several levels. It could be done at the source—i.e., the institution that houses the specimen and serves the data fundamentally. It could—and has—been done at the level of what Dr. Page terms "intermediate portals" (e.g., VertNet). Or it could be done by GBIF, although GBIF has not shown any incentive in that direction. Regardless of at what level it gets done, what we do emphasize is that the changes be repatriated back to the original level… we have now made this latter point more clearly.

I'm being slightly facetious, but a serious point is whether GBIF has the tools or the motivation to fix errors in data - to date they've resisted doing this. This paragraph "This insight can guide time investment in biodiversity informatics initiatives. Analyses such as those we have developed identify immediately the limiting dimensions of DAK usability, thereby focusing immediate investments of time and energy. The clearest signal from our analyses is that detailed and well-documented georeferencing is a crucial aspect of biodiversity informatics, although particular situations can and will differ significantly from this generality. In other senses, some biodiversity informatics activities—although important clearly—may not pay off in usable information as immediately. For instance, basic digitization is a major emphasis in the field, and is important for collections management, but digitization in an institutional framework that does not foster data sharing will not improve and increase the availability of information for science and policy." seems to conflate two separate issues, namely data quality (or fitness for use) and data being open.

We get Dr. Page's facetiousness, and both we and he have argued strenuously from within GBIF that GBIF should pay greater attention to data quality, including georeferencing. And we all have been frustrated that—for whatever reason—GBIF has not jumped into this challenge. It is disappointing, to be honest. We cannot force these changes, but we can provide yet another statement of the problem… whoever decides to take on the challenge, but someone *indeed needs to take on the challenge.*

As to conflating data quality and data openness, we note that our wording was somewhat confusing, such that Dr. Page might have thought that such was our intention. Not true … and we have adjusted the wording in that paragraph to make it clear that the sentence about data openness is now clearly not a follow-on to the preceding sentences.

The authors seem to be arguing in favour of georeferencing and against digitising more specimens, which seems odd.

Not at all. We simply make the point that lack of high-quality georeferencing appears to be the biggest bottleneck in making more data immediately usable. We used an unfortunate conjunction, however, such that we see how this linkage could be perceived in our wording. It has now been corrected, and we hope that our real point is now more clear.

Given that georeferencing emerges as their main concern, what, if anything, can we do to tackle this issue. Is the current model of georeferencing adequate for the task? What about approaches such as Cardoso et al. "A Gazetteer for Biodiversity Data as a Linked Open Data Solution" https://doi.org/10.1109/WETICE.2014.19 ?

Thanks for pointing out this paper to us … it is interesting and relevant and we have now cited it in the manuscript. However, we are not convinced that this paper is the place in which to discuss and decide next technologies and platforms for georeferencing. We have conducted and presented an analysis of data leakage from biodiversity data resources, and we arrive at some concrete conclusions about which parts of the biodiversity informatics data flow are most prone to leakage. The question of how *to plug those leaks and improve the overall biodiversity informatics data flow is a separate issue… in some cases, we can imagine mass data processing to add georeferences to records; in other cases, we can imagine crowdsourcing as the best approach to the challenge, or a community-based solution like what VertNet did. In sum, we do not see this paper as the place for a detailed discussion of these points, as they involve much customization and exploration that is beyond the scope of the present manuscript.*

The authors state in several places that no global species names authority lists for plants were available. What about The Plant List, or the iPlant Taxonomic Name Resolution Service http://tnrs.iplantcollaborative.org/index.html?

Well, we checked both of those sources, and I just rechecked them, and neither was available for download as a standalone dataset, which is what would be necessary for the analyses that we were doing. It may be possible that a smarter person could use the TNRS web-based facility, but we were not able to. We have made every effort to be clear about what we have and have not done in our analyses.

It is unclear what the authors mean by "GBIF taxonomic name filtering". GBIF processes the data it receives and may reinterpret the names that data providers send to GBIF. I don't see why this would only affect a subset of searches - presumably it affects all searches that use a taxon name?

As we had stated in the sentence to which Dr. Page refers, our experience is that there is no name filtering when the queries are not via name. That is, if you download all records of Harpia harpyja*, you get records with that name. However, if you download all records from the New World, you get name variants that are in fact records of that species. Why? We have no idea, but that is our experience, and we stated it explicitly as such.*

The criterion for fully usable geographic data seems stiff, and gives the impression that huge amounts of data are unusable. I suspect the majority of users do not look at coordinatePrecision and coordinateUncertaintyInMeters, if only because the values reported often don't make much sense (at least in my experience). Furthermore, we are never completely ignorant of uncertainty, it is possible to infer uncertainty from things such as coordinate precision, and the verbatim locality description (e.g., is the locality a country, or a more precise regions within a country?).

We get that our criteria for full usability of the place information are stiff and perhaps hard to satisfy. And indeed, Dr. Page is correct that not all niche modelers or other biodiversity informatics analysts take these steps, but that does not eliminate the fact that they should *take these steps in the process of pretty much any application that requires mapping. Techniques exist by which to incorporate such information in analyses, and it is completely clear that not taking it into account compromises the quality of the resulting analyses. Peterson has used such information extensively in analyses of North American vertebrates … they are maximum radii of uncertainty, measured in meters, and they can be used to filter out uncertain localities (e.g., Peterson, A.T., Lash, R.R., Carroll, D.S., Johnson, K.M., 2006. Geographic potential for outbreaks of Marburg hemorrhagic fever. American Journal of Tropical Medicine & Hygiene 75, 9-15.) or they can be incorporated explicitly into analyses (e.g., Kissling, W.D., Carl, G., 2008. Spatial autocorrelation and the selection of simultaneous autoregressive models. Global Ecology and Biogeography 17, 59-71.). We have added text and a reference to make this point more clearly.*

Likewise, there are no measures of confidence for temporal or taxonomic data, would it not be consistent to either (a) require confidence measures for all kinds of data or (b) for none? There are clearly differences in georeferencing practices between different data providers, and between taxonomic groups (as evidenced by the authors treating the Brazilian Virtual Herbarium dataset as having many records with full information despite their low degree of geographic precision). My sense is that many plant datasets are grid-based not point-based, so the the point-radius method that typifies vertebrate datasets might not be an appropriate yard-stick by which to assess georefencing for plants.

As we stated in response to an earlier comment from Dr. Page, what exists for georeferencing is a metadata standard. We have used a 4-level confidence-and-completeness measure for all three of the data dimensions (place, time, taxon), but we just have more information for place, thanks to the MaNIS metadata protocol. So we are not treating place any differently from time or from taxon in our analyses.

It is true that the Brazilian Virtual Herbarium data "look" good because they are mostly endowed with uncertainty information. That, however, is the point—for better or for worse, those data are able to be evaluated for use because they carry full documentation of their quality. Other data may be just as good or bad, but they are not documented, and as such cannot even be assessed.

Many plant datasets may be grid-based, but we are focused on herbarium specimen records. To our knowledge, the locational data for these specimens are point-based, as each had a textual country-state-locality "georeference," which in some cases had been translated into geographic coordinates. As such, although some other plant datasets may have grid systems, we did not see evidence of these data being so cast. And regardless, a grid-based georeferencing system can be translated into points and uncertainties pretty easily.

"To provide a broader perspective on these data leaks" seems a weak justification for including the Brazilian Virtual Herbarium and VertNet. The justification for their inclusion seems more that the authors are involved in those projects.

This assertion is odd. There is a perfectly good reason to include the two big datasets… to get an idea about the generality of the patterns that we identified in our selected examples! This was stated in the original manuscript, and still holds. In fact, simply for the record, Wieczorek and Canhos were invited to participate as coauthors specifically *because we wanted to include that information. It is probably best not to attempt to read peoples' minds when one is attempting to interpret something… and the simplest explanation is likely the best… a view of the generality of the situation would be highly informative in this analysis!*

The claim "yet data leakage certainly is more frequent as the age of the specimen increases" is not tested - it is simply asserted. Given that the authors have information on time, presumably they can test this assertion?

We did some preliminary exploration of this point, and conclude that the situation is relatively complex, and we will prefer to develop this point in a separate treatment. That is, the following three figures are trends in proportion of records usable (gray part of bar, status level 2 or 3) versus not usable (black part of bar, status 0 or 1) by decade, for the Harvard Herbarium, for time, place, and taxon…

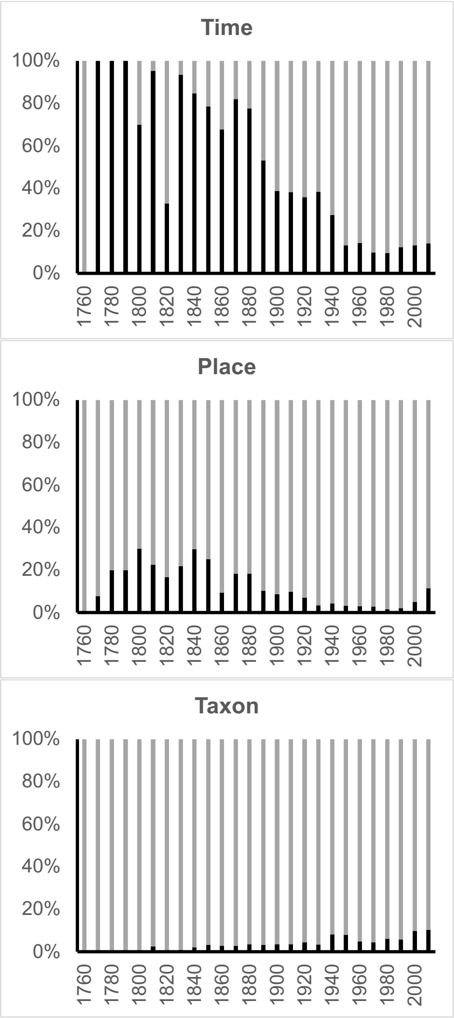

Quite clearly, temporal information shows greater "leakage" in older specimens, and place information shows similar trends, if more subtle. However, taxon information, if anything, shows the opposite trend, with increasing numbers of records showing incomplete taxonomic information in more recent decades. As such, we prefer to develop this point in a future contribution, in which it can be treated in greater detail. For the moment, we have added some verbiage to the Discussion, but we defer full treatment.

The value of a separate protocol at https://doi.org/10.17504/protocols.io.kebctan seems moot given that without the exact datasets and scripts used by the authors, coupled with the use of proprietary software (Microsoft Access) it will be a challenge to reproduce the study. It will also be a challenge because the data used is not provided (either as downloads, or as DOIs for GBIF downloads).

The separate protocol is a requirement of the journal, and was not our idea! It is there, if one wishes to refer to it, and can be ignored if one does not wish to. The datasets are now provided, so that one can reproduce our analyses. Our analyses were not scripted… Peterson is not endowed with those abilities, and so did the work the old-fashioned way.

However, we are intrigued by the criticism of our use of Microsoft Access for analysis. Does this criticism mean that a paper should be rejected if the authors used ArcMap instead of QGIS? Wow! Note, please, that all of the datasets are given in ASCII format, so as to be universally readable; however, the fact that we used proprietary software for some of the analyses, while lamentable, does not seem like an appropriate basis for criticizing the work.

Helen Hardy

As noted above, I am not comfortable with 'loss' as a descriptor in this context. 'Leakage' is well explained but 'loss' to me goes a step further than what is described here, which is essentially incompleteness.

We agree entirely with this critique, and have avoided the use of the term "loss" in the paper now.

Related to this, the discussion section of this paper briefly touches on a wide range of issues around the reasons for data incompleteness and how these could be addressed. I would like to see these addressed more systematically, which I believe could be done without disproportional effort and I think is important earlier in the paper in setting the context for the analysis done. Specifically, I would like to see: (a) Earlier in the paper, a short description of data sources, setting out the differences between observational and specimen data (and thus clarifying that the latter is the scope of this study)

We have added a sentence to the Introduction in response to this suggestion, in which we introduce the specimen-observation contrast earlier in the manuscript.

(b) An early summary of the causes and categories of data incompleteness. The diagram and description of leakage, while good, does not spell out the differences between data which was missing at source (e.g. not recorded at the point of collection); data which has been subject to

random or systematic error at some point during its curation or digitisation; and data which has been deliberately excluded from a process of digitisation but may be added later (e.g. requires expert review to parse - see also (c) below). This is separate to the concept of rescueability as defined here.

Verbiage regarding this point has now been added to the Introduction, to introduce these ideas earlier in the manuscript.

There are also points raised briefly in the discussion about causes of data incompleteness, e.g. the challenges of historic collections, which could be made sooner and slightly more fully. This would also give context to the reference in the discussion to 'inevitable' data loss which currently jars with the main conclusions of the paper.

We removed the use of the world "inevitably," and added clearer wording to make this point less ambiguous.

(c) Most importantly, in the context of the key insight here about geo-referencing, it would be helpful to include a recognition (e.g. in the discussion) that digitisation without full geo-referencing is often a first stage (for instance owing to lack of resources or perhaps expertise/tools) which may or could be followed by full geo-referencing at a later date. I would like if anything to see the conclusion about geo-referencing brought out even more fully / strongly - perhaps to recommend that all of those engaged in creating and releasing biodiversity data from specimens take account of the relevance of full geo-referencing to the kinds of science discussed (I can envisage using the information in this paper to support a business case for georeferencing resources, for instance). The Authors may wish to refer to Nelson et al, 'Five task clusters that enable efficient and effective digitization of biological collections' doi: 10.3897/zookeys.209.3135

The Nelson et al. paper is now cited, and we have added verbiage to the Discussion to speak to this important point.

I am also not able to access the protocol file, so am not clear to what extent the full data and outcomes of this study are available or could be replicated - for that reason I have selected 'major' revision, however this may in practice be more minor.

The full data are now provided, so that the work can be replicated exactly. As to the protocol file, it was created, and we were able to access it from Tanzania (!)… it is at http://dx.doi.org/10.17504/protocols.io.kebctan.
