# Supplementary material for: Data Leakage and Loss in Biodiversity Informatics
Source: Biodivers Data J. 2018 Nov 7;(6):e26826. doi: 10.3897/BDJ.6.e26826 (PMC6235996; doi:10.3897/BDJ.6.e26826)
Supplement: Supplementary material 2 — Appendix: Summary Tables [file bdj-06-e26826-s002.pdf]

Appendix: Data Tables

Tables of data underlying figures 2 and 3. In each case, the primary biodiversity data held by the particular collection at the particular institution are separated into information that is absent (no information), partial, rescuable, or full, with respect to dimensions of time, taxonomic name, and place, as well as in terms of name x place jointly, and in terms of all three dimensions jointly.

BIRDS

MCZ	Time	Place	Name	NamePlace	All 3
No information	29543	1276	13	1288	29658
Partial information	31	32916	305	33140	16821
Rescuable information	1	241698	7463	242070	231914
Full information	288809	42494	310603	41886	39991

AMNH	Time	Place	Name	NamePlace	All 3
No information	349	1127	0	1127	1431
Partial information	69	4287	0	4287	4416
Rescuable information	0	125552	38981	125613	125200
Full information	132582	2034	94019	1973	1953

KSTC	Time	Place	Name	NamePlace	All 3
No information	51	70	8	8	55
Partial information	1	1700	14	81	63
Rescuable information	0	0	146	1681	1652
Full information	1718	0	1602	0	0

NCSM	Time	Place	Name	NamePlace	All 3
No information	1293	407	0	407	1360
Partial information	1192	1772	44	1804	2362
Rescuable information	0	18912	299	18880	17369
Full information	18606	0	20748	0	0

PSM	Time	Place	Name	NamePlace	All 3
No information	245	181	1	182	338
Partial information	466	246	2	248	646
Rescuable information	0	4617	1092	5472	5280
Full information	24849	20516	24465	19658	19296

KU	Time	Name	Place	NamePlace	All 3
No information	9329	116	8312	8314	10508
Partial information	1786	271	6	253	1797
Rescuable information	5	839	45582	45994	43284
Full information	110919	120813	68139	67478	66450

HERBARIA

HARVARD	Time	Place	Name	NamePlace	All 3
No information	185759	21433	7163	26681	191024
Partial information	126936	45278	23498	60529	148382
Rescuable information	0	488199	0	467700	215504
Full information	242215	0	524249	0	0

GHANA	Time	Place	Name	NamePlace	All 3
No information	3890	0	51	51	3931
Partial information	8750	19679	1573	20807	25208
Rescuable information	5	58539	7202	57360	48979
Full information	65573	0	69392	0	0

CMN	Time	Place	Name	NamePlace	All 3
No information	2945	267	1076	1342	4195
Partial information	3014	0	0	0	2940
Rescuable information	0	119136	2222	118061	112268
Full information	113444	0	116105	0	0

INPA	Time	Place	Name	NamePlace	All 3
No information	2899	7458	19952	26802	28792
Partial information	8790	0	0	0	8056
Rescuable information	0	244283	40482	224939	214893
Full information	240052	0	191307	0	0

MPEG	Time	Place	Name	NamePlace	All 3
No information	4839	52	5989	6028	10620
Partial information	8630	0	0	0	8418
Rescuable information	0	155088	32432	149112	136102
Full information	141671	0	116719	0	0

MSC	Time	Place	Name	NamePlace	All 3
No information	1183	212	616	746	1770
Partial information	4380	0	0	0	4304
Rescuable information	0	65114	2457	64580	59252
Full information	59763	0	62253	0	0

UA	Time	Place	Name	NamePlace	All 3
No information	1615	200	55	255	1792
Partial information	1330	0	0	0	1275
Rescuable information	0	41260	508	41205	38393
Full information	38515	0	40897	0	0

USF	Time	Place	Name	NamePlace	All 3
No information	1048	7844	138	7970	8865
Partial information	4667	0	0	0	4184
Rescuable information	0	132737	1163	132611	127532
Full information	134866	0	139280	0	0
